# Efficient Breast Cancer Classification Network with Dual Squeeze and Excitation in Histopathological Images

**DOI:** 10.3390/diagnostics13010103

**Published:** 2022-12-29

**Authors:** Md. Mostafa Kamal Sarker, Farhan Akram, Mohammad Alsharid, Vivek Kumar Singh, Robail Yasrab, Eyad Elyan

**Affiliations:** 1 National Subsea Center, Robert Gordon University, Aberdeen AB10 7AQ, UK; 2 Institute of Biomedical Engineering, University of Oxford, Oxford OX1 2JD, UK; 3 Department of Pathology and Clinical Bioinformatics, Erasmus Medical Center, 3015 GD Rotterdam, The Netherlands; 4 Department of Electrical Engineering and Computer Science, Khalifa University, Abu Dhabi P.O. Box 127788, United Arab Emirates; 5 Independent Researcher, Belfast BT9 5GD, UK; 6 School of Computing Science and Digital Media, Robert Gordon University, Aberdeen AB10 7AQ, UK

**Keywords:** breast cancer, histopathology, convolutional neural network, dual squeeze, excitation mechanism

## Abstract

Medical image analysis methods for mammograms, ultrasound, and magnetic resonance imaging (MRI) cannot provide the underline features on the cellular level to understand the cancer microenvironment which makes them unsuitable for breast cancer subtype classification study. In this paper, we propose a convolutional neural network (CNN)-based breast cancer classification method for hematoxylin and eosin (H&E) whole slide images (WSIs). The proposed method incorporates fused mobile inverted bottleneck convolutions (FMB-Conv) and mobile inverted bottleneck convolutions (MBConv) with a dual squeeze and excitation (DSE) network to accurately classify breast cancer tissue into binary (benign and malignant) and eight subtypes using histopathology images. For that, a pre-trained EfficientNetV2 network is used as a backbone with a modified DSE block that combines the spatial and channel-wise squeeze and excitation layers to highlight important low-level and high-level abstract features. Our method outperformed ResNet101, InceptionResNetV2, and EfficientNetV2 networks on the publicly available BreakHis dataset for the binary and multi-class breast cancer classification in terms of precision, recall, and F1-score on multiple magnification levels.

## 1. Introduction

Breast cancer is the most common type of cancer in women worldwide, with a high mortality rate independent of the economic status of an individual country. Among women, it can be experienced in different age groups, where risk goes higher as they get older. Every year around 2.4 million cases of cancer are diagnosed, out of which approximately one-fifth succumb to death [1,2], making it a public health concern. One of the main reasons for such a high mortality rate among women is due to an inaccurate and late diagnosis of breast cancer [3].

Currently, there are many medical imaging methods for detecting breast tissue abnormalities, including mammography, ultrasound, magnetic resonance imaging (MRI), and computed tomography (CT). These imaging modalities try to capture the spatial structure of the cancer tissue. However, it is hard to differentiate the cancer tissues from the normal ones when the breast density is high [4]. Moreover, these modalities cannot provide the underline features on the cellular level to understand the cancer microenvironment, which makes them unsuitable for breast cancer subtype classification studies. A pathological diagnosis is considered a *gold standard* for an accurate identification [5] and subtype classification of the cancer tissue. For that, a biopsy is taken from the breast cancer region, then hematoxylin and eosin (H&E) staining is used to obtain a cellular and morphological underlying structure of extracted cancer tissue in the form of an H&E whole slide image (WSI). However, the precise interpretation of H&E WSIs always creates many challenges. The clinical diagnosis is subjective that varies from one pathologist to another. Furthermore, breast cancer cell identification is very labor-intensive, time-consuming, and prone to error. Finally, in developing countries, trained pathologists are scarce, and facilities are inadequate. In these circumstances, it is less likely for there to be an automated solution to perform clinical diagnoses based on histopathological images.

**Figure 1:**
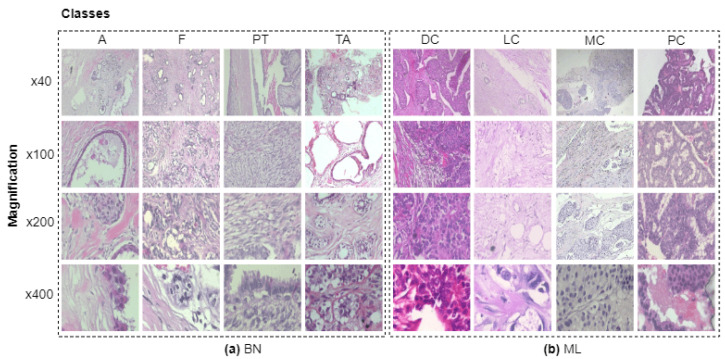
Illustration of H&E-stained breast tumour patch examples extracted from the different magnifications of whole slide image (WSI). Note that (**a**) benign and (**b**) malignant correspond to the BN and ML, respectively. It contains four histological subtypes of benign breast tumours: adenosis (A), fibroadenoma (F), phyllodes tumour (PT), and tubular adenona (TA); and four malignant tumours (breast cancer): carcinoma (DC), lobular carcinoma (LC), mucinous carcinoma (MC), and papillary carcinoma (PC). These sub-categorized breast tumours are distinct in their visual, textural patterns and color features.

**Motivation:** Breast cancer subtyping is a demanding task that plays a crucial role in clinical diagnosis [6]. Both intra- and inter-class heterogeneity in the underlying cell morphology, color, and texture of the microenvironment of the cancer tissue makes cancer subtyping quite challenging [6]. Figure 1 shows four sub-classes of benign and malignant cancer tissue. From the visual inspection, it is evident that each row corresponds to the specific magnification (i.e., 40×, 100×, 200×, and 400×) that follows varying textural and color patterns of individual classes.

In recent years, deep learning-based convolutional neural networks (CNNs) have achieved remarkable success in histopathology image analysis [5,7]. These methods extract high-level cellular, color, and textural image features from H&E WSI in an automated manner that plays a crucial role in cancer classification. To date, numerous traditional and deep-learning-based methods have been proposed to classify abnormalities in breast tissue [8,9]. However, the generalizability of these methods under different image magnifications is still a challenge that makes the cancer tissue sub-typing even harder. To manually identify the various pathological cellular and morphological features is time-consuming and error-prone. Therefore, an automated solution is needed to save pathologists’ time and reduce human error.

**Contribution:** In this paper, we propose a method that incorporates fused mobile inverted bottleneck convolutions (FMB-Conv) and mobile inverted bottleneck convolutions (MBConv) with a dual squeeze and excitation (DSE) network to accurately classify breast cancer tissue into eight subtypes using histopathology images. We utilize a pre-trained EfficientNetV2 [10] network as a backbone to extract enriched features. This network incorporated a Fused-MBConv block that substitutes depth-wise conv3×3 and expands conv1×1 with smaller 3×3 kernel sizes [10]. The Fused-MBConv layers foster the network training speed by increasing a few trainable parameters. We incorporated a DSE [11] block that combines the spatial and channel-wise squeeze and excitation layers and highlights important low-level and high-level abstract features. We perform binary and multi-class classifications using BreakHis dataset [12]. The binary class includes classifying breast cancer into benign or malignant. However, in the multi-class task, benign and malignant cancer tissues are subdivided into eight subtypes (four for benign and four for malignant). The benign breast cancer tissues are divided into adenosis, fibroadenoma, phyllodes tumour, and tubular adenoma. Whereas the malignant breast cancer tissues are categorized into carcinoma, lobular carcinoma, mucinous carcinoma, and papillary carcinoma. We performed extensive experiments comparing recent state-of-the-art methods with various image magnifications. The experimental results demonstrated that the method presented in this study is robust in classifying breast cancer tissue into binary and multi-class categories outperforming the state-of-the-art methods with significant margins.

The remainder of this paper is organized as follows. Section 2 discusses the related work that attempted to solve the breast cancer classification problem in histopathological images. Section 3 includes a detailed description of the dataset and the proposed methodology. Section 4 provides the experimental results under various settings. Finally, we conclude our findings and suggest future research work in Section 5.

## 2. Related Works

In recent years, numerous architectures have been proposed to solve the breast cancer classification problem. Below we present and discuss the ensemble and some multiple-CNN-based methods, transfer learning-based methods, and other approaches in the literature.

### 2.1. Ensemble and Multiple CNN-Based Methods

There is extensive research in the literature on using multiple CNNs together to classify breast cancer in histopathological images [7]. Kassani et al. [13] used an ensemble of deep-learning networks to classify histopathological biopsy images. Their ensemble-based approach makes use of VGG16 [14], MobileNet [15], and DenseNet [16] networks to extract rich features. The authors combined breast tumour features of all models rather than using features of individual models only. This approach overcomes the limitations of each classification method and provides the strength of multiple CNNs to capture a variety of distinct features. The suggested ensemble approach improved the classification accuracy but required high computational resources with more time requirements to process all the deep models in the prediction. This issue poses challenges for adaptation to clinical practice.

Instead of using features from several different neural networks, Gupta et al. [17] used multi-layered features arising from different levels of a fine-tuned DenseNet network. The authors recommended a solution to combine the multi-layers features that captured the lower spatial and higher level global structural features to boost the classification performance in the sequential framework. They achieved a classification accuracy of 96.71% at a magnification of 200× on the BreakHis dataset.

Zhu et al. [18] assembled compact CNNs to classify breast cancer in histopathology images. The authors employed the hybrid CNN design that involves local and global model branches with the same CNN architecture. The authors combined features from these two branches and applied local voting to extract robust features. Moreover, they ignored the unwanted channels or features with the suggested squeeze-excitation-pruning mechanism and achieved higher classification results.

Ukwuoma et al. [19] also used a combination of CNNs to classify breast cancer histopathology images by extracting global features and spatial information from regions of interest. Aljuaid et al. [3] used a combination of multiple pre-trained CNNs, including ResNet18 [20], ShuffleNet [21], and Inception-V3 [22] networks. The authors also applied transfer learning to their BreakHis dataset at various magnification levels. They enhanced the classification performance through the data augmentation techniques, such as flipping, rotations, and translations. The authors achieved a classification accuracy of 97.81% and 99.70% for binary and multi-class, respectively, using the ResNet method. All the existing methods utilized the various CNNs architectures and combined them to extract rich feature information from the histopathology images but limited them to the clinical translation due to the design of the complex structure that requires higher computational resources with a longer time.

### 2.2. Transfer Learning-Based Methods

Transfer learning methods rely on using networks that have been previously trained on a similar or adjacent task. The parameter weights of those networks are frozen. The last (few) layer(s) are replaced or changed according to the needs of the task at hand. This fact explains how transfer learning could be used for both binary breast cancer classification and multi-class breast cancer classification [23]. As mentioned earlier, ref. [3] used a number of CNNs in a transfer learning-based framework. The approach of Gupta et al. [17] can fall under this category as well due to relying on fine-tuning pre-trained CNNs. However, Xie et al. [23] also applied the transfer learning technique to train InceptionV3 and InceptionResNetV2 for breast cancer classification into binary and multi-class categories. InceptionV3 and InceptionResNetV2 have been pre-trained on ImageNet [24]. The authors applied the transfer learning techniques using the InceptionResNetV2 network in an auto-encoder to analyse unseen images through a K-means clustering algorithm, which clusters the same class images together. They applied the data augmentation technique and provided an increment in the classification performance. The authors include flipping images around the x−y axes and rotating clockwise with an angle between 90° and 180°. Motlagh et al. [1] fine-tuned ResNet-50 and ResNet-152 networks to classify the histopathology images into benign or malignant classes. The authors also utilized these networks to classify them into multiple sub-classes. The transfer-learning method permits leveraging feature representations from an existing ImageNet pre-trained network. However, in some cases, it is not very helpful since medical domain features are entirely different from natural images. This creates an issue in generalizing the model performance under various vendor scanner images to achieve robust results.

### 2.3. Other Approaches

Apart from above discussed approaches, other methods exist for breast cancer classification tasks. Curriculum learning-based approaches usually follow a prescribed regimen of training samples for the model to encounter. Typically, but not necessarily, it would range from ‘easy’ examples to ‘complex’ ones. Mayouf et al. [25] employed a curriculum-learning strategy on the BreakHis dataset called curriculum incremental deep learning. The authors trained the model with images of a specific magnification level. The weights of that model were then used to initialize the weights of the model when training with histopathology images at a larger magnification level. The process starts with 40×, 100×, 200×, and finally, 400× magnification. The incrementation comes in the form of increasing magnification levels. Training starts with the lowest magnification level and increases steadily till the maximum magnification level is incorporated towards the end of training. Seo et al. [26] recommended a primal-dual multi-instance support vector machine (SVM) to segment the breast tissue comprising the cancer cells. The proposed approach outperformed the traditional SVM-based method. Hao et al. [27] proposed a method that employed the fusion of DenseNet201 deep semantic features and three-channel GLCM features for the breast cancer classification task in histopathology images. The authors achieved a classification accuracy of 96.75% with 40× magnification on the BreakHis dataset.

Han et al. [6] used a structured deep learning model to perform breast cancer multi-classification from histopathological images, taking into account the different magnification levels of the benign and malignant images in the BreakHis dataset.

## 3. Materials and Methods

### 3.1. Dataset

In this study, we used the publicly available Breast Cancer Histopathological Image Classification (BreakHis) dataset [12]. It includes 82 patients with breast tumour tissue. This dataset consists of 9109 microscopic images with multiple magnifications, such as 40×, 100×, 200×, and 400×. Specifically, BreakHis incorporated 2480 benign and 5429 malignant samples stored in PNG format. It followed the 3-channel RGB with an average resolution size of 700×460 pixels. Table 1 illustrates the detailed description of the BreakHis dataset. We split the dataset into training, validation, and test sets with a ratio of 70%, 10%, and 20%, respectively. It should be worth noting that we evaluated the model performance on an independent test set, which was not included in either training or validation.

### 3.2. Model Architecture

**Figure 2:**
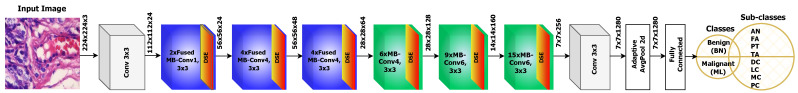
Overview of the proposed classification model.

In this work, we use a dual squeeze and excitation (DSE) [11] block that incorporates fused mobile inverted bottleneck convolutions (Fused MB-Conv) and mobile inverted bottleneck convolutions (MBConv) to identify breast cancer in histopathology images. The proposed method’s schematic diagram is shown in Figure 2. The DSE block consists of spatial and channel ‘squeeze and excitation (SE)’ mechanisms. The spatial SE techniques obtained a global contextual representation by selectively aggregating the context in accordance with a spatial SE map by developing pertinent semantic features that can benefit both groups and improve intra-class semantic consistency. In contrast, the channel SE can emphasize class-dependent feature mappings and discriminatively support a feature enhancement that the convolution layers are unable to produce. Thus, combining these two SE processes can improve the feature representation of intra-class differences in channel maps. Initially, the input histopathology images patch of 224×224×3 *I* fed into one standard convolutional (*Conv*) layer with a kernel size of 3×3 and stride of 2. The feature map of the $F^{Conv_{kxk}}$ is defined as,

(1) $$F^{Conv_{k\times k}}\left(x,y\right)=\sum_{r}\sum_{c}I\left(r,c\right)K\left(x-r,y-c\right)$$

where *r* represents the row index, *c* represents the column index, *I* is the input image, *K* is the convolutional kernel, and k×k represents the size of the convolutional kernel, which can be either 3×3 or 1×1 depending on what part of the architecture the convolution is occurring in.

Afterwards, three FusedMB−Conv’s (2×FusedMB−Conv1_3×3, 4×FusedMB−Conv4_3×3, and 4×FusedMB−Conv4_3×3) blocks with *DSE* is used. The FusedMB−Conv operation relies on a 3×3 convolution followed by a *DSE* block and then finally with a 1×1 convolution, as defined below:

(2) $$F^{FusedMB}=F^{Conv_{1\times 1}}\left(DSE\left(F^{Conv_{3\times 3}}\left(F_{input}^{FMB}\right)\right)\right)+F_{input}^{FMB}$$

where $F_{input}^{FMB}$ is the input feature to the Fused MB-Conv block. The DSE block can be represented by Equation (3):

(3) $$DSE=F_{Input}^{DSE}⊗AP(F^{Conv_{1\times 1}}\left(F_{relu}\left(F^{Conv_{1\times 1}}\left(FC_{sigmoid}\right)\right)\right)+F_{Input}^{DSE}⊗F^{Conv_{1\times 1}}\left(FC_{sigmoid}\right))$$

where $F_{Input}^{DSE}$ represents the input to the DSE block, AP represents the average pooling operation, and $F_{relu}$ represents the operations an activation function of ReLU.

Subsequently, three MB-Conv (6 × MB-Conv4_3 × 3, 9 × MB-Conv6_3 × 3, and 15 × MB-Conv6_3 × 3) with *DSE* are applied. The MB-Conv operation uses a 1 × 1 convolution as the first step, followed by a 3 × 3 depthwise convolution, a *DSE* block, and a final 1 × 1 convolution, as defined below:

(4) $$F^{MB}=F^{Conv_{1\times 1}}\left(DSE\left(Depth^{conv}\left(F^{Conv_{1\times 1}}\left(F_{input}\right)\right)\right)\right)+F_{input}$$

Finally, a final 3 × 3× convolution with AP and a fully connected (*FC*) layer is used to finalize the feature map to classify the input image as Benign (BN) or Malignant (ML), and also with the sub-types of BN (AN, FA, PT, and TA) and ML (DC, LC, MC, and PC), respectively.

### 3.3. Loss Function

Since the BreakHis dataset [12] is unbalanced, selecting the appropriate loss function is crucial when training deep learning models. Thus, we trained our proposed model using the class-balanced (CB) focal loss function [28]. The CB loss offers a weighting factor to address the challenge of deep network training with unbalanced data. Contrarily, the focal loss (FL) [29] adds a scaling factor to the sigmoid cross-entropy loss to reduce the associated loss for successfully identified cases and concentrate on challenging examples. For a given image I with ground-truth G∈{1,2,…,c}, where *c* is the number of all classes, the class probabilities $C_{p}={\left[p_{1},p_{2},\ldots ,p_{c}\right]}^{⊤}$, where $p_{k}\in \left[0,1\right]\;\forall \;k$, denote $p_{k}^{t}=\mathrm{sigmoid}\left(I_{k}^{t}\right)=1/\left(1+\exp\left(-I_{k}^{t}\right)\right)$ is calculated by the model, the FL is denoted as follows:

(5) $$L^{\mathrm{FL}}\left(I,G\right)=-\sum_{k=1}^{c}{\left(1-p_{k}^{t}\right)}^{\sigma }\;\log\left(p_{k}^{t}\right)$$

The following is a description of the final CB focal loss:

(6) $$L^{\mathrm{CB}}\left(I,G\right)=-\frac{1-\gamma }{1-\gamma^{n_{G}}}\sum_{k=1}^{c}{\left(1-p_{k}^{t}\right)}^{\sigma }\;\log\left(p_{k}^{t}\right)$$

where $\left(1-\gamma \right)/\left(1-\gamma^{n_{G}}\right)$ is the weighting factor of the loss function with the hyperparameter γ∈[0,1], σ∈[0.5,2] and $n_{G}$ is the number of images in the ground-truth class *G*.

## 4. Experimental Results and Discussion

### 4.1. Training Details

During the training procedure, we resized the original hematoxylin and eosin (H&E) patches to 224×224 pixels. All the images were normalized by estimating the mean and standard deviation. We applied data augmentation that included the 30-degree rotation and horizontal and vertical flips with a probability of 0.5. The applied data augmentation techniques help to increase the sample size, enable the narrowing of the semantic feature gap, and provide additional discriminative features to improve the classification performance. We incorporated the SGD optimizer with a learning rate of 0.001 and trained the network with four batch sizes. Note that all the networks followed the same hyperparameter settings and were trained with 100 epochs. Note that the images between the benign and malignant classes are imbalanced. Therefore, we used the class-specific weighting mechanism that applied to the loss function. This allows it to have a greater weight focus on the classes with fewer samples.

**Computational Setup:** We developed the models using the FastAI platform and used the PyTorch neural network library with 11 GB GPU memory on Nvidia RTX2080Ti.

### 4.2. Evaluation Metrics

To assess the proposed model classification performance, we used three metrics precision (*PR*), recall (*RE*), and *F*1-*score* (*F*1). The formulations of these metrics are provided in the below equations.

(7) $$Precision\left(PR\right)=\frac{TP}{TP+FP}$$

(8) $$Recall\left(RE\right)=\frac{TP}{TP+FN}$$

(9) $$F1\mathrm{-s}core\;\left(F1\right)=\frac{2TP}{2TP+FP+FN}$$

where *TP*, *TN*, *FP*, and *FN* refer to the true positives, true negatives, false positives, and false negatives, respectively.

### 4.3. Results

Table 2 shows the binary class classification results of the proposed model compared with three state-of-the-art methods (ResNet101 [20], InceptionResnetV2 [22], and EfficientNetV2 [10]) for diagnosing benign and malignant types in histopathology images. We reported the results for individual slide magnification, including 40×, 100×, 200×, and 400×. The experimental results confirm that the proposed model has better classification performance in all the metrics than the second-best EfficientNetV2. Specifically, it obtained a 3% increment on the 400× magnification than EfficientNetV2. Note that the proposed model combined the DSE mechanism that led to the improvement in the classification performance. The proposed model captures the fine details of the cell structure through the DSE mechanism that highlights the most relevant cell-related features. However, on the 200× magnification, InceptionResNetV2, EficientNetV2, and the proposed approach yield very similar scores in the range of 98–99%.

**Figure 3:**
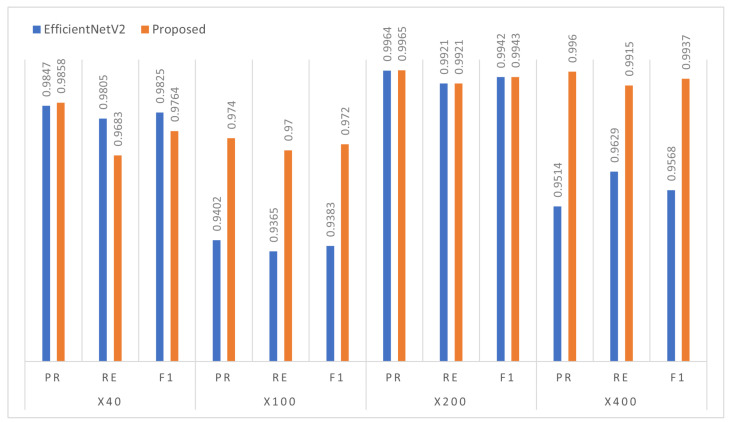
Illustration of the binary class classification performance achieved by the proposed model compared to the EfficientNetV2 network under multiple magnification settings.

**Figure 4:**
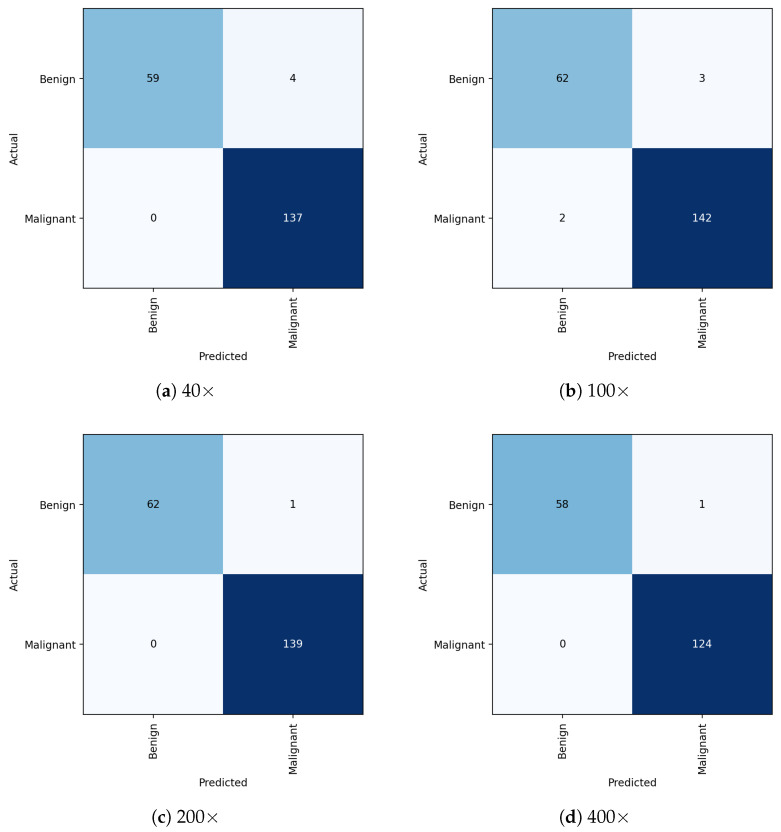
Illustration of the binary class confusion matrix obtained through the proposed model on the test set. Note that (**a**–**d**) refer to the 40×, 100×, 200×, and 400× magnifications, respectively.

**Figure 5:**
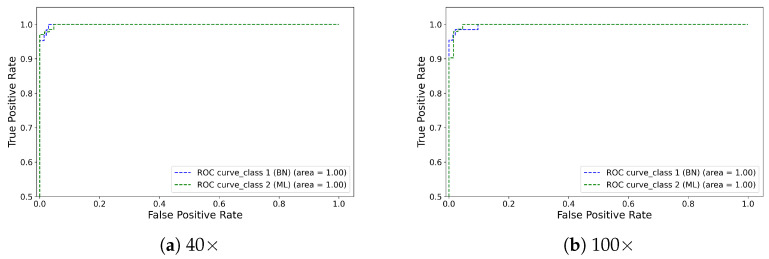

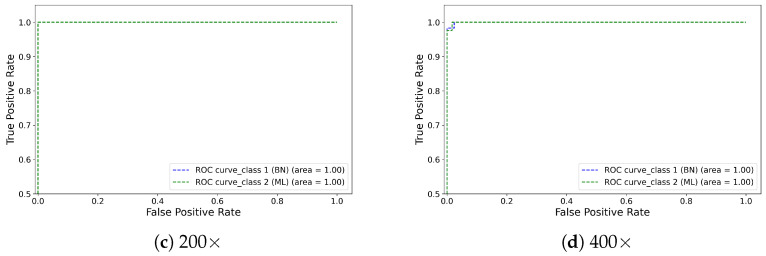
Illustration of the ROC curve obtained through the proposed model on the test set. Note that (**a**–**d**) refer to the 40×, 100×, 200×, and 400× magnifications, respectively.

Conclusively, as we increased the magnification level, the proposed model showed increasingly promising results (see Figure 3) by accurately classifying the benign and malignant tumour cells. In this binary classification task, each class of tumour cells has different textural patterns that help the CNN-based proposed approach to extract those key features and accurately classify them. We found that adding the FMB-Conv and MBConv helped to enhance the feature representation, and DSE provided more attention to the relevant, targeted cell features. Figure 4 illustrates the confusion matrix for the proposed model evaluated on the test set at different magnification levels. It is evident that the proposed approach precisely classifies the two distinct classes with lower misclassification errors. Figure 5 demonstrates the ROC curves for each benign and malignant class at all four magnifications. We found that the proposed model received an area under the curve (AUC) score of 100 for each class, including benign and malignant. Using the class-balanced (CB) focal loss function in which we computed the weights of each class and provided more weightage to the classes with fewer samples allowed us to overcome the model overfitting issue, thereby improving breast cancer classification performance into benign and malignant classes.

**Figure 6:**
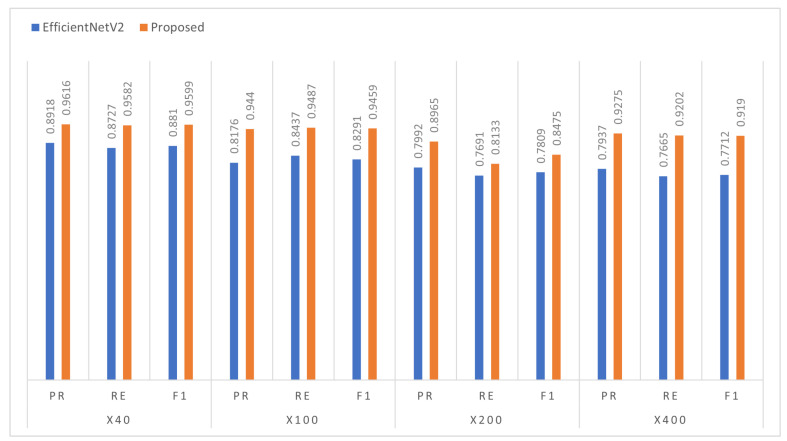
Illustration of the proposed model multi-class classification performance improvement respective to EfficientNetV2.

Table 3 demonstrates the classification results for eight classes. The combined eight classes included the main benign and malignant tumours and were categorized into four sub-classes each. The benign breast tumours include adenosis, fibroadenoma, phyllodes tumour, and tubular adenona. The four malignant tumours include carcinoma, lobular carcinoma, mucinous carcinoma, and papillary carcinoma. We evaluated the efficacy of the proposed model under different magnifications (40×, 100×, 200×, and 400×) against the recent state-of-the-art methods. With 40×, the proposed model attained a significant improvement with 7% in terms of PR, RE, and F1 scores compared to the EfficientNetV2. Whereas, ResNet101 and IncepetionResNetV2 scored lower performances. As the magnifications increased to 100×, the proposed model precisely determined the pattern of multi-class cells increased (see Figure 6) and accurately discriminated the different cells with a prominent margin of 10% over EfficientNetV2 and InceptionResnetV2. On 200×, the proposed model followed a similar significant improvement as 100×. However, we have noticed the great classification performance by the proposed model with 400× where the cancer cells are zoomed and more distinct. Since each class of cells has its own unique morphology and textural patterns, identifying those features is necessary to make the correct prediction. Therefore, the convolutional layers with DSE mechanisms provide more distinct features that help the model accurately classify the textural patterns of multi-class cells and yield more than a 12% improvement in all metrics than the rest.

**Figure 7:**
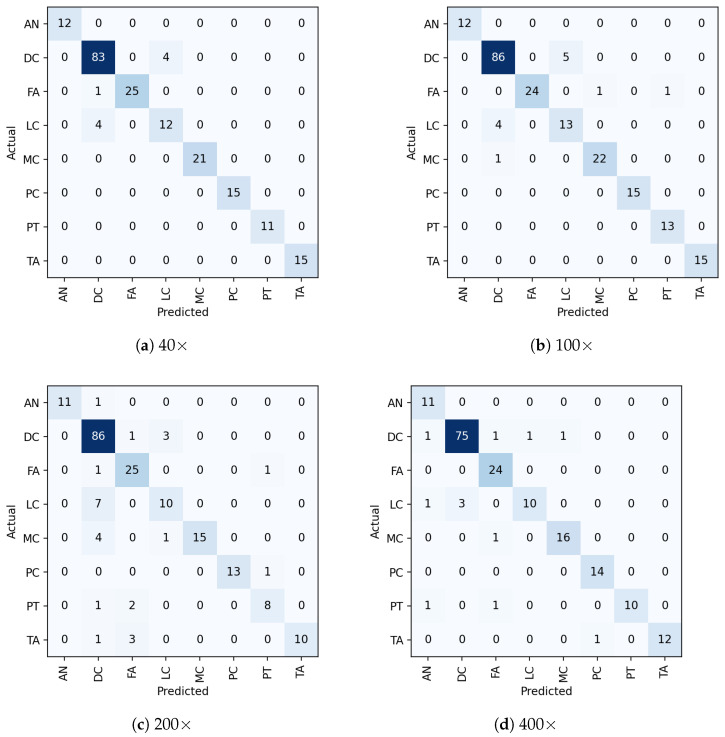
Illustration of multi-class confusion matrix obtained through the proposed model on the test set. Note that (**a**–**d**) refer to the 40×, 100×, 200×, and 400× magnifications, respectively.

**Figure 8:**
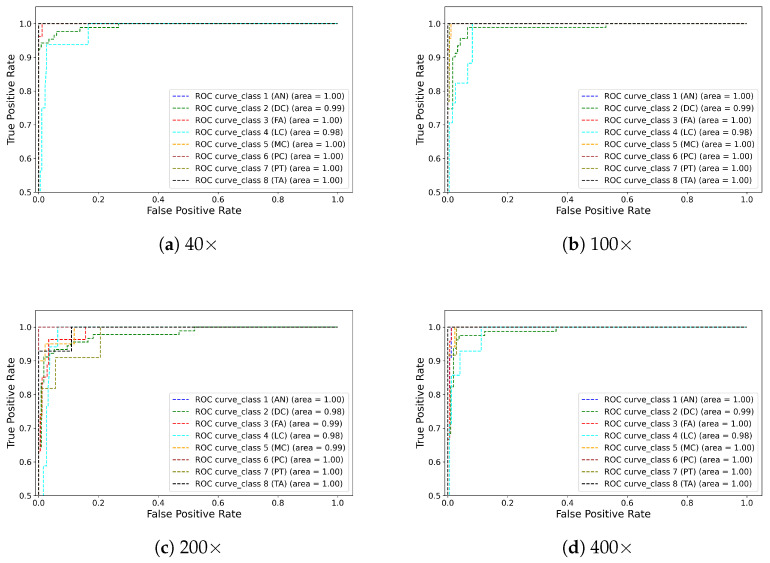
Illustration of the ROC curve of eight classes obtained through the proposed model on the test set. Note that (**a**–**d**) refer to the 40×, 100×, 200×, and 400× magnifications, respectively.

Figure 7 shows the eight class classification results generated by the proposed method. The best classification result is shown diagonally for each class. As can be seen, all the sub-classes are classified well, with only very few misclassifications to other classes at different magnifications. Figure 8 presents the class-wise AUC score generated by the proposed model on the test set. All eight classes have achieved an AUC score of more than 99%. All the quantitative and qualitative measures demonstrate that the proposed model is capable of precisely classifying the images into multiple sub-classes. We noticed that some samples were misclassified to other classes caused due to the presence of imaging artefacts and needed more training examples to add variability so that the issue can be overcome, enhancing the classification performance.

### 4.4. Discussion and Limitation

**Figure 9:**
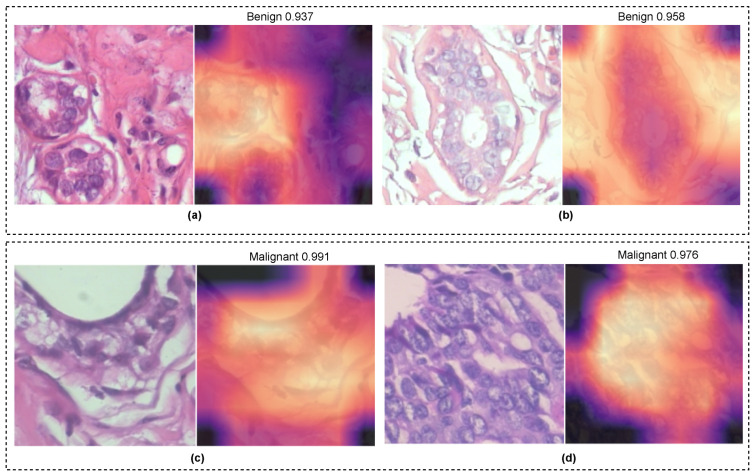
GradCam visualisation of the proposed model for H&E-stained breast tumour patch examples of benign (**a**,**b**) and malignant (**c**,**d**) classes.

We developed an efficient deep-learning-based classification model to classify breast cancer with sub-classes in H&E. Our experimental findings suggested that the proposed approach is more robust than the other state-of-the-art methods. For a fair comparison, we compared the proposed method with the three CNN-based methods consisting of ResNet101 [20], InceptionResnetV2 [22], and EfficientNetV2 [10]. These compared methods yield acceptable results but are not good enough to show the generalizability with binary and multi-class problems. The proposed model incorporated the MBConv with a dual squeeze and excitation (DSE) layer into the EffciientNetV2. The addition of an attention mechanism allows the model to capture more relevant feature representations, such as cell structure, textural patterns, and morphology information, and ignore the unwanted background pixels. This model exhibited the capability to classify images of multiple magnifications and achieve better classification results with 400×. Figure 9 provided the GradCam visualisation of the proposed model for benign and malignant classes. It is clearly evident that the model accurately highlighted the targeted cells and ignored the rest. The model achieved a very high confidence rate in predicting the tumour patches. Conclusively, the introduced approach is more robust and provides higher classification results that could help to make a better diagnosis. We found that our model has one limitation. It struggles to accurately classify a few samples where imaging artefacts, such as blurriness and improper cell boundaries, are present.

## 5. Conclusions

In this paper, we proposed a CNN-based breast cancer classification method for binary (benign and malignant) and multi-class (adenosis, fibroadenoma, phyllodes tumour, tubular adenoma, carcinoma, lobular carcinoma, mucinous carcinoma, and papillary carcinoma) tasks. The proposed method incorporates fused mobile inverted bottleneck convolutions (FMB-Conv) and mobile inverted bottleneck convolutions (MBConv) with dual squeeze and excitation (DSE) layers into a pre-trained EfficientNetV2 to classify using histopathology images. We perform binary and multi-class classifications using the BreakHis dataset [12]. Empirical results demonstrated the robustness of the proposed method in classifying breast cancer tissue into binary and multi-class categories. It outperformed the state-of-the-art methods with significant margins. In future work, we would like to validate the proposed model on other cancer types such as colon, bladder, lung, melanoma, etc.

## Figures and Tables

**Table 1:** A breakdown of the publicly available breast cancer classification *BreakHis* dataset. This dataset contains multiple image magnifications defined for benign and malignant categories.

Dataset	Magnification	Benign	Malignant	Total
BreakHis [12]	40×	652	1370	1995
	100×	644	1437	2081
	200×	623	1390	2031
	400×	588	1232	1820
	Total of Images	2480	5429	7909

**Table 2:** Classification performance of the proposed model compared with three state-of-the-art diagnoses of breast cancer benign and malignant types in histopathology images. Note that ‘PR’ stands for ‘Precision’, ‘RE’ stands for ‘Recall’, and ‘F1’ represents the ‘F1-score’. The best significant result is highlighted in bold.

Models	Performance Metrics											
	Magnifications											
	40×			100×			200×			400×		
	PR	RE	F1	PR	RE	F1	PR	RE	F1	PR	RE	F1
ResNet101	0.8761	0.8395	0.8543	0.8389	0.836	0.8374	0.9052	0.9121	0.9085	0.8467	0.837	0.8415
InceptionResnetV2	0.9732	0.9811	0.977	0.9561	0.9673	0.9614	0.9808	0.9849	0.9828	0.9329	0.929	0.9309
EfficientNetV2	0.9847	**0.9805**	**0.9825**	0.9402	0.9365	0.9383	0.9964	**0.9921**	0.9942	0.9514	0.9629	0.9568
**Proposed (EfficientNetV2 + DSE)**	**0.9858**	0.9683	0.9764	**0.9740**	**0.9700**	**0.9720**	**0.9965**	**0.9921**	**0.9943**	**0.996**	**0.9915**	**0.9937**

**Table 3:** Classification performance of the proposed model compared with three state-of-the-art models to diagnose eight types of benign and malignant breast cancer in histopathology images. The most significant results are highlighted in bold.

Models	Performance Metrics											
	Magnifications											
	40×			100×			200×			400×		
	PR	RE	F1	PR	RE	F1	PR	RE	F1	PR	RE	F1
ResNet101	0.7743	0.7808	0.7698	0.7486	0.7463	0.7388	0.7255	0.6895	0.7052	0.7937	0.7665	0.7712
InceptionResnetV2	0.8684	0.8336	0.8485	0.8385	0.8324	0.8319	0.7767	0.7582	0.7609	0.7835	0.6956	0.7229
EfficientNetV2	0.8918	0.8727	0.881	0.8176	0.8437	0.8291	0.7992	0.7691	0.7809	0.7937	0.7665	0.7712
**Proposed (EfficientNetV2 + DSE)**	**0.9616**	**0.9582**	**0.9599**	**0.944**	**0.9487**	**0.9459**	**0.8965**	**0.8133**	**0.8475**	**0.9275**	**0.9202**	**0.919**

## Data Availability

https://web.inf.ufpr.br/vri/databases/breast-cancer-histopathological-database-breakhis/ (accessed on 12 October 2022).
